# Evaluation of Microwave Ablation in 4T1 Breast Tumor by a Novel VEFGR2 Targeted Ultrasound Contrast Agents

**DOI:** 10.3389/fonc.2021.690152

**Published:** 2021-07-20

**Authors:** Xiaoyu Li, Shujun Xia, Ri Ji, Weiwei Zhan, Wei Zhou

**Affiliations:** ^1^ Department of Ultrasound, RuiJin Hospital, School of Medicine, Shanghai Jiaotong University, Shanghai, China; ^2^ Department of Ultrasound, RuiJin Hospital/Lu Wan Branch, School of Medicine, Shanghai Jiaotong University, Shanghai, China

**Keywords:** vascular endothelial growth factor receptor 2 targeted, breast cancer, contrast enhanced ultrasound, ultrasound contrast agent, microwave ablation

## Abstract

**Objectives:**

A novel ultrasound contrast agent (UCA) VEGFR2-targeting iron-doped silica (SiO_2_) hollow nanoparticles (VEGFR2-PEG-HSNs-Fe NPs) was prepared and applied in microwave ablation for breast cancer to investigate its value in the evaluation of effectiveness after tumor ablation.

**Methods:**

VEGFR2-PEG-HSNs-Fe NPs were prepared by using nano-SiO_2_, which was regarded as a substrate and etched by ferrous acetate, and then modified with anti-VEGFR2 antibody. Laser confocal microscope and flow cytometry were used to observe its main physicochemical properties, and biological safety was also investigated. After the xenograft tumor was treated with microwave ablation, the extent of perfusion defect was evaluated by ultrasound by injecting VEGFR2-PEG-HSNs-Fe NPs.

**Results:**

The average particle size of VEGFR2-PEG-HSNs-Fe was 276.64 ± 30.31 nm, and the surface potential was −13.46 ± 2.83 mV. *In vitro*, the intensity of ultrasound signal increased with UCA concentration. Good biosafety was performed in *in vivo* and *in vitro* experiments. The enhanced ultrasound signal was detected in tumors after injection of VEGFR2-PEG-HSNs-Fe NPs, covering the whole tumor. The lesions, which were incompletely ablated, presented as contrast agent perfusion at the periphery of the tumor, and contrast enhanced ultrasound (CEUS) was performed again after complementary ablation. It was confirmed that all the lesions were completely ablated.

**Conclusion:**

Nano-targeted UCAs VEGFR2-PEG-HSNs-Fe NPs had good biosafety and ability of specific imaging, which might be used as a contrast agent in CEUS to evaluate the efficacy of tumor ablation.

## Introduction

Minimally invasive thermal ablation technology for tumor has attracted more and more attention due to its unique advantages and good curative effect (1–3). Microwave ablation (MWA) is a relatively new technology, which has been proven to be a safe and effective minimally invasive treatment for tumor. At present, MWA has been widely used in the treatment of benign and malignant tumors of various organs, such as thyroid, liver, kidney, and lung. The clinical data showed that good clinical effects were obtained (4–7). However, some literature showed that non-lethal thermal stimulation could promote the proliferation and invasion of tumor cells (8, 9). This also meant that incomplete ablation of cancer tissue may promote tumor progression. Therefore, in order to ensure that residual tumor can be detected in time after ablation, postoperative imaging was necessary to evaluate whether the ablation was complete or not (10, 11). However, the local hyperechoic area was formed immediately after ablation due to the vaporization of tumor tissue. The high echo was not conducive to the conventional ultrasound observation of the ablation effect and the determined the ablation range, so it was difficult to accurately measure the ablation area with unclear edge. Thus, the value of conventional ultrasound in evaluating the ablation range immediately after ablation was limited (12, 13).

Contrast enhanced ultrasound (CEUS) was a real-time dynamic imaging technology. The non-linear harmonic echo could be generated by the injection of ultrasound contrast agents (UCAs), which could better evaluate the microcirculation perfusion of tumor tissue. It had been widely used in the evaluation of range after ablation of various tumors (14–16). However, at present, the conventional UCAs were mainly micron scale, which lacked the ability of targeting and could not display the ablation range continuously and accurately. Moreover, due to the rapid regression time, the residual lesions could not be displayed continuously, and multiple injections may be required. Thus, the application of conventional UCAs in ablation was limited.

Targeted UCAs could specifically concentrate on the target area and enhance the imaging effect of lesion (17, 18). At the same time, due to removal of the limitation of micron scale, nano UCAs had stronger penetration and could realize extravascular development (19). Vascular endothelial growth factor receptor 2 (VEGFR2) was highly expressed on the surface of tumor neovascular endothelial cells. Through the specific binding of UCAs targeting VEGFR2 with VEGFR2 site, it could bind to vascular endothelial cells and enhance ultrasonic echo signal, which could specifically display tumor vessels. Moreover, the nano UCAs could reach the outside of the blood vessel through the endothelial space. Due to lack of mature capillary network and poor lymphatic reflux, the metabolic capacity decreased in new tumor tissue. Nanoparticles can accumulate in the interstitial space (20) for a long time, which could enhance the display of tumor tissue. Based on these, we proposed an assumption that nano-targeted UCAs might have important clinical significance for guiding ablation therapy and evaluating ablation efficacy.

Therefore, the purpose of this paper was to prepare a new targeted nano UCA VEGFR2-PEG-HSNs-Fe NP to study its physicochemical properties and evaluate the feasibility of its application *in vivo*. 4T1 breast tumor-bearing mice models were established to simulate the microenvironment of VEGFR2 positive new tumor tissue. After microwave ablation, the prepared targeted contrast agent was used to evaluate the curative effect and judge whether the tumor was completely ablated. On one hand, we could explore the imaging effect of the new VEGFR2 targeted nano UCAs in mice models. On the other hand, we could judge its value in evaluating the curative effect of tumor ablation.

## Material And Method

### Material

Silane-polyethylene glycol (PEG)-COOH (molecular weight 2,000) was obtained from Ponsure Biological (Shanghai, China). Iron(II) acetate was provided by Titan Technology (Shanghai, China). Hydroxylamine hydrochloride, 1-ethyl-(3-dimethylaminopropyl) carbodiimide (EDC) hydrochloride, and N-hydroxysuccinimide (NHS) were purchased from Aladdin Chemistry (Shanghai, China). Anti-VEGFR2/KDR antibody and P-phycoerythrin (PE)-conjugated rabbit antimouse VEGFR2 monoclonal antibody were obtained from Sino Biological (Beijing, China).

### Preparation of VEGFR2-PEG-Fe-HSNs

SiO_2_ NPs were prepared by a previously reported (21) modified Stöber method. The mixture of 1.5 ml deionized water, 50 ml ethanol, and 2.5 ml ammonia was heated to 55°C. Tetraethyl orthosilicate, 2 ml, was dripped into the mixture at 8 ml/h and stirred for 4 h. Then, SiO_2_ NPs were obtained after centrifugation (10,000 rpm, 5 min). HSNs-Fe NPs were synthesized by hydrothermal method (22, 23). Briefly, 30 mg SiO_2_ NPs and 200 mg iron(II) acetate were added in 18 ml deionized water. The mixture was transferred to an oven at 180°C for 24 h. After cooling, HSNs-Fe NPs were collected by centrifugation (9,000 rpm, 5 min).

In order to get VEGFR2-PEG-HSNs-Fe NPs, we first modified the surface of HSNs-Fe NPs with silane-PEG-COOH. Silane-PEG-COOH, 30 mg, and 10 mg HSNs-Fe were suspended in 20 ml mixture of ammonia and ethanol (v:v 1:1). After shake incubation for 24 h at room temperature, PEG-HSNs-Fe NPs were obtained by centrifugation (8,000 rpm, 4 min). Secondly, anti-VEGFR2 antibodies were attached to PEG-HSNs-Fe NPs by carbodiimide method. An 8 mg EDC, 12 mg NHS, and 30 mg PEG-HSNs-Fe were dissolved in 20 ml PBS and stirred for 4 h to activate -COOH. Centrifugation (10,000 rpm, 5 min) was repeated three times in order to remove the excess unreacted material. Subsequently, 20 µl anti-VEGFR2 antibody was added into 20 ml PBS solution mixture (1 mg/ml) and incubated for 12 h. Finally, VEGFR2-PEG-HSNs-Fe NPs were prepared by centrifugation (10,000 rpm, 5 min) for four times.

### Characterization of NPs

Morphology and structure of HSNs-Fe NPs were observed by transmission electron microscopy (TEM; JEM-2100F; Jeol, Tokyo, Japan). Size distribution of HSNs-Fe NPs was tested by dynamic laser scattering (DLS; ZS3690; Malvern Instruments, Malvern, UK). Confocal laser-scanning microscopy (CLSM; TCS SP5 II; Leica, Wetzlar, Germany) was used to observe the antibody modification and targeting ability to cells of VEGFR2-PEG-HSNs-Fe NPs. US imaging ability of VEGFR2-PEG-HSNs-Fe NPs was investigated by ultrasound machine (MyLab 90; Esaote, Genoa, Italy), and the appropriate imaging concentration was selected for the *in vivo* experiments.

### Cell Targeting Ability of VEGFR2-PEG-HSNs-Fe NPs

#### Western Blot Detected VEGFR2 Protein Expression

RIPA was added into human umbilical vein endothelial cells (HUVECs) and 4T1 cells for 3 to 5 min. The cells were dissolved by repeated blowing in ice bath for 30 min and centrifugation (12,000 rpm, 10 min) for three times. SDS loading buffer was added and boiled at 100°C for 10 min. A 10% separation gel and 5% concentrated gel were prepared for the samples, and 12 μl protein was added into each sample hole. Electrophoresis was carried out in 80 V constant pressure mode, followed by membrane transfer in 120 V for 120 min. After blocking, VEGFR2 antibody (ACTIN as internal reference) was diluted at the concentration of 1:1,000 and incubated at 4°C overnight. Images were developed by electrochemiluminescence (ECL) and analyzed after being incubated with the second antibody for 2 h.

#### Cells Targeting Ability

The specific targeting ability *in vitro* was tested by HUVEC and 4T1 cells in two ways: qualitative observation by CLSM and quantitative detection by flow cytometer (FCM; Beckman Coulter, Brea, CA, USA). Four groups were designed: simple cells, targeted competition, non-targeted, and targeted groups. In the targeted competition group, 100 μl (PE) VEGFR2-PEG-HSNs-Fe was added to the pretreated cells, which were incubated with VEGFR2 antibody for 30 min. Targeted and non-targeted groups were added 100 μl (PE) VEGFR2-PEG-HSNs-Fe and PEG-HSNs-Fe, respectively. After incubation for half an hour, cells were used in the next two experiments: (1) cells were treated with 1 ml 4% paraformaldehyde for 20 min, followed by 200 μl DAPI staining for 5 min. Images were collected by CLSM after washing three times with PBS. (2) Cells were digested with trypsin and centrifuged (1,500 rpm, 5 min) three times. Cell suspension was obtained after re-suspended with 2 ml PBS and sent to FCM to quantify the fluorescence intensity of the cells.

### Biosafety of VEGFR2-PEG-HSNs-Fe NPs

#### Apoptosis

In this part, three repeated holes were designed in each group. Different concentrations (50, 100, 200 μg/ml) of VEGFR2-PEG-HSNs-Fe NPs 100 μl and PBS 100 μl were added to the corresponding cells and cultured for 24 h. After centrifugation (1,500 rpm, 5 min), 5 × 10^5^ cells were collected and re-suspended with 100 μl binding buffer, 5 μl PI standing solution, and 5 μl annexin V-FITC were added and incubated at room temperature for 10 min. Following addition of 400 μl binding buffer, all cells were sent to FCM for detection.

#### Blood Indexes

All animal experiments were carried out under the guidelines formulated by China Animal Health Committee and approved by the ethics committee and Animal Care Committee of Ruijin Hospital, School of medicine, Shanghai Jiaotong University. All animals were provided by vital River Laboratory Animal Technology Company (Beijing, China).

According to the injection dose of VEGFR2-PEG-HSNs-Fe NPs, healthy mice were divided into four groups: 10 mg/kg, 20 mg/kg, 30 mg/kg, and PBS. Peripheral blood was collected through the orbit at 24 h after injection and centrifuged (1,000 rpm, 10 min) to obtain the supernatant (50 μl/tube) for testing.

#### Establishment of Mouse Model

4T1 cells were cultured in a mixture of DMEM medium (90%), fetal bovine serum (9%), and penicillin/streptomycin (1%). After digestion and centrifugation, a cell suspension with a concentration of 1 × 10^8^/ml was prepared. Subsequently, the suspension was mixed with an equal volume of Matrigel under ice-water bath. Subcutaneously, 200 μl of the mixture was subcutaneously injected into the back near the right forelimb of the female BALB/C nude mice at 4–6 weeks. After that, the tumor was observed every other day. Mice bearing 4T1 breast tumor were used for *in vivo* imaging and ablation efficacy evaluation experiments until the tumor volume reached 150–200 mm^3^.

#### Pathological Examination of Breast Tumor

Briefly, all tumor tissues were dehydrated and soaked in xylene I, II, and paraffin, followed by cutting and deparaffinization with xylene. Then, tumor tissues were fixed, dehydrated, embedded, and sectioned. Then, the sections were dyed with hematoxylin for 30 min, faded in 1% ethanol, and washed with distilled water for 30 s in sequence. After immersing in saturated lithium carbonate solution for 2 min, the tissue sections were re-stained with 0.5% eosin ethanol for 1–3 min, followed by rinsing again. Subsequently, the sections were dehydrated in 80, 90, 95, and 100% ethanol for 5 min, and then were transferred into xylene I and xylene II for 15 min respectively. Finally, the samples were sealed with neutral resin before being observed under the microscope.

The expression of VEGFR2 protein was detected by immunohistochemistry and double staining. After dewaxing and dehydration, the tissue sections were put into EDTA antibody repair solution. Anti-VEGFR2 antibody was diluted with 1:100 and incubated with sections at 4°C for one night. Soon afterwards the second antibody diluted by 1:300 was added and cultured for another night. Then, the slices were incubated with PE labeled anti-CD31 antibody at a dilution of 1:50 for 1 h at room temperature. After 10 min of DAPI staining, the sections were washed and the images were collected by fluorescence microscope.

#### Immunology Assay

Tumor-bearing nude mice (4 weeks, 50% female, 50% male, n = 3 each) were used in this part. VEGFR2-PEG-HSNs-Fe NPs were injected *via* tail vein at dose of 30 mg/kg in the experimental groups, while the control group was uninjected. According to the time points (0.5, 1, 12, 24 h) after injection, blood was collected. Detection and quantification of immune factors were completed by Shanghai Huaying Biological Co., Ltd. Microsoft Excel software was used to draw the heat map to display the difference of factors.

### US Imaging *In Vivo*

In *in vivo* imaging experiments, the commonly used ultrasound instrument MyLab 90 with LA 522 linear probe was selected. B mode and CEUS dual imaging mode were used to observe in real-time, and images were collected. CEUS mode parameters were set as follows: probe frequency = 7.5–10 MHz, MI = 0.10, depth = 37 mm, gain = 44%, power = 9%. All laboratory animals were pre-anesthetized by intraperitoneal injection of 5% chloral hydrate (0.1 ml/10 g) before the experiment.

#### Specific US Imaging

The specific imaging experiments were divided into four groups: targeted, competition, non-targeted, and SonoVue control group. Mice in the corresponding groups were injected with VEGFR2-PEG-HSNs-Fe, anti-VEGFR2 antibody, and VEGFR2-PEG-HSNs-Fe, PEG-HSNs-Fe and SonoVue. The competition group was pre-injected with 20 μl of VEGFR2 antibody solution diluted to 200 μl with PBS, followed by VEGFR2-PEG-HSNs-Fe injection. Each experimental mouse was injected with 200 μl of the corresponding UCAs *via* tail vein. The injection concentration of SonoVue was 1 mg/ml and that of the rest three groups was 20 mg/ml.

The nude mice bearing tumors were completely anesthetized and fixed on a constant temperature heating plate, and the transplanted tumors were fully exposed. After applying excessive coupling agent, the tumor was scanned in the transverse and longitudinal sections, and the largest section was selected for imaging. Images of the tumor area at 1, 10, and 30 min after the contrast injection were collected. The intensity of ultrasound signal in CEUS mode was quantified by Image J (1.48v).

#### Evaluation Ablation of CEUS

Tumor size was evaluated in the transverse and longitudinal section. The largest section of the tumor was selected as the plane of ablation path. Length (a), width (b) and height (c) of the tumor maximum section were recorded, and the tumor volume was calculated according to the following formula, (V) : V = a × b × c × 0.52.

The water-cooled microwave antenna was connected with MTC-3 system (Vison-China Medical Devices R&D Center, China) and cold circulation device. Under real-time ultrasound guidance, a 19G microwave antenna was inserted into the tumor. When the tip of the antenna exceeded 1 mm of the tumor, cold circulation and fixed ablation started at the power output of 35 W. Ablation time was adjusted according to the tumor size. Tumor tissue would be vaporized by the energy of ablation. When the hyperechoic area just covered the tumor, ablation was stopped. After the gas subsided, UCAs were injected *via* the tail vein to evaluate the perfusion of the ablation area. If any perfusion of UCAs was observed, we adjusted the position of microwave antenna to re-ablate until no perfusion was observed in the ablation area. Then, operation time was recorded. The area of perfusion defect was measured, and the volume was calculated.

### Statistical Analysis

Quantitative data were expressed as mean ± SD. Analysis of variance (ANOVA) was used to determine the significance of differences in multiple groups using SPSS software version 13.0 (SPSS Inc; Chicago, IL, USA). P <0.05 was considered statistically significant.

## Result And Discussion

### Characterization of NPs

**Figure 1:**
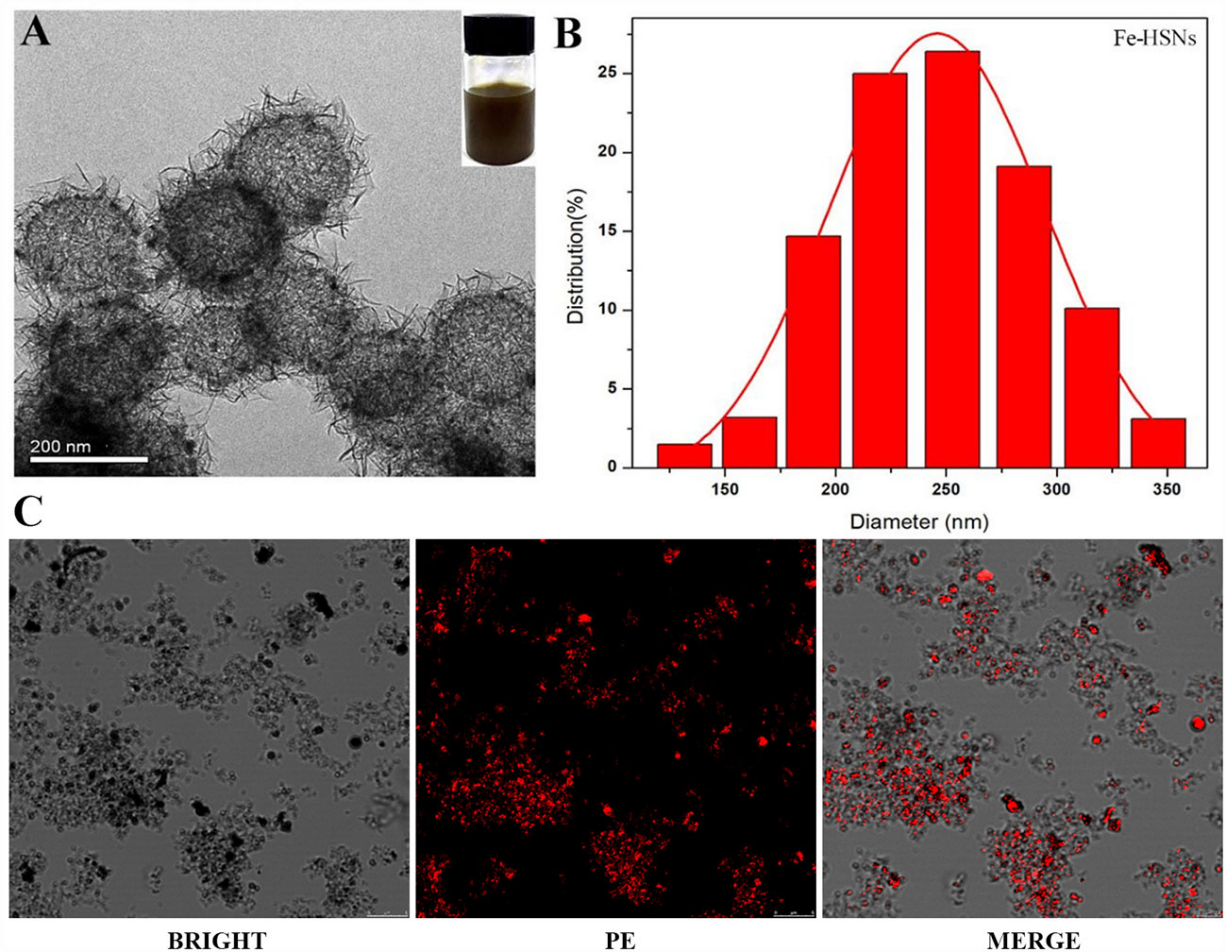
Characteristic of NPs. **(A, B)** TEM and diameter distribution image of Fe-HSNs. **(C)** LSCM images of VEGFR2-PEG-HSNs-Fe. VEGFR2-PEG-HSNs-Fe modified with luciferin PE by laser excitation showed red fluorescence.

Referring to the method in the previous literature (22, 23), HSNs-Fe NPs were synthesized by hydrothermal method. HSNs-Fe NPs aqueous solution was black with good dispersion. TEM results (**Figure 1A**) showed that HSNs-Fe NPs were hollow spheres with rough surface and uniform particle size. The average size was 245.68 ± 23.58 nm (**Figure 1B**), and the surface potential was −32.21 ± 3.72 MV. Taken silane-PEG-COOH as the bridge, anti-VEGFR2 antibody was conjunct to HSNs-Fe NPs to prepare VEGFR2-PEG-HSNs-Fe NPs. Their mean size was 276.64 ± 30.31 nm, and surface potential was −13.46 ± 2.83 MV. Size and surface potential change of VEGFR2-PEG-HSNs-Fe NPs might result from the coupling of various media including silane-PEG-COOH, anti-VEGFR2 antibody, and NPs.

In order to further evaluate the modification of antibody, we prepared VEGFR2-PEG-HSNs-Fe with fluorescein PE labeled anti-VEGFR2 antibody and observed NPs directly by LSCM. PE showed red fluorescence under laser excitation of LSCM. The LSCM images (**Figure 1C**) showed that VEGFR2-PEG-HSNs NPs were not only evenly distributed in the field of vision, but also showed a clear hollow spherical structure. A large number of red fluorescence could be observed, and most of the fluorescence positions overlapped with NPs. These results suggested the successful preparation of VEGFR2-PEG-HSNs NPs and good antibody modification effect.

The purpose of preparing nano targeted UCAs in this study was that it could not only specifically bind to VEGFR2 protein of tumor neovascular endothelial cells, but also pass through the vascular endothelial space into the tumor tissue. As the results showed above, VEGFR2-PEG-HSNs-Fe NPs with an average diameter of 200 nm could achieve increased penetration and retention (24–26). As previously reported, NPs could pass through 380–700 nm vascular endothelial cell space (27) and continuously accumulate in tumor tissue. Therefore, the retention time can be extended so as to achieve ultrasound imaging (19).

**Figure 2:**
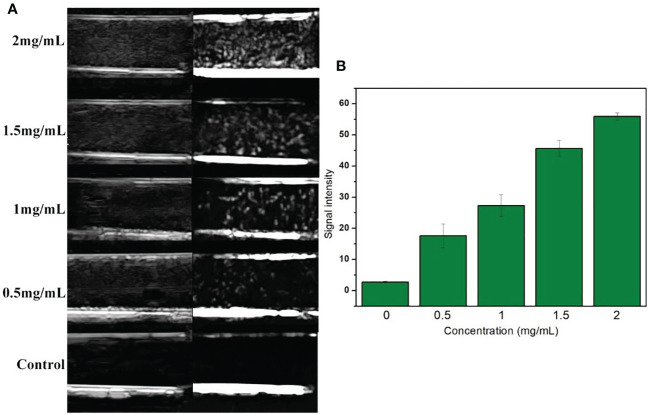
**(A)** US images of VEGFR2-PEG-HSNs-Fe with different concentrations; **(B)** signal intensity of VEGFR2-PEG-HSNs-Fe with different concentrations in CEUS mode.

Generally speaking, the smaller the size of UCAs, the weaker was the echo. Although the literature had confirmed the development capability of many nano targeted CAs, whether the VEGFR2-PEG-HSNs-Fe NPs could also show satisfactory imaging effect still needed to be confirmed. *In vitro* ultrasound images (**Figure 2A**) with VEGFR2-PEG-HSNs-Fe NP solution showed hyperechoic spot in B and CEUS modes, while PBS solution in control group showed no echo. Even at low concentration of 0.5 mg/L, VEGFR2-PEG-HSNs-Fe NPs still had a significant hyperechoic signal. Further, the ultrasonic signal strength increases with the increase in VEGFR2-PEG-HSNS-Fe-NPs concentration (**Figure 2B**). These results indicated that VEGFR2-PEG-HSNs-Fe NPs had good ultrasonic imaging ability. This also provided powerful conditions for the experiments *in vivo*.

### Targeting Capability *In Vitro*

**Figure 3:**
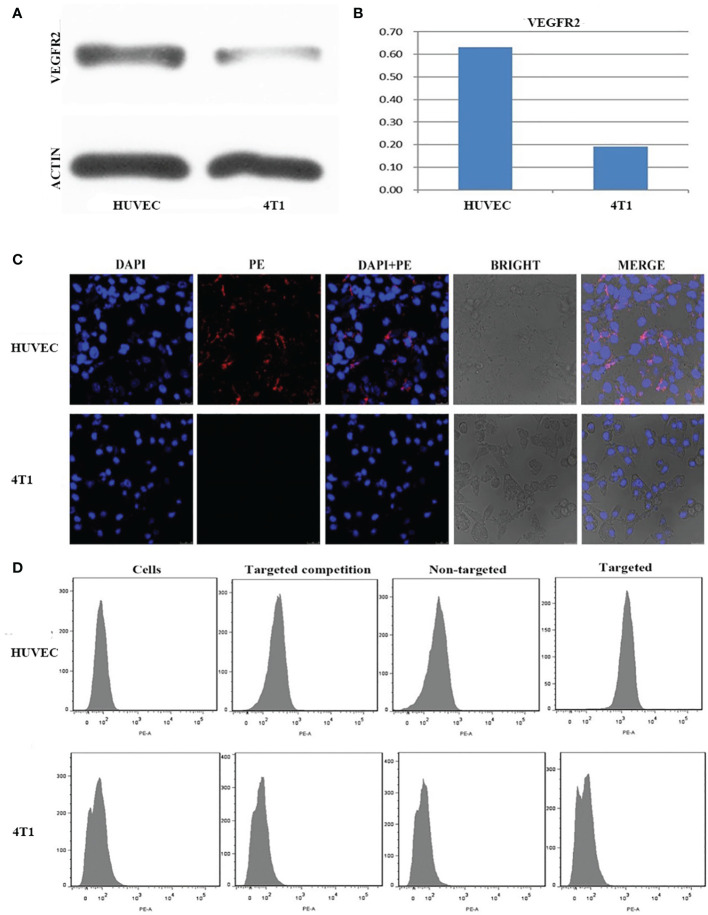
**(A)** Western-blot images and quantitative analysis **(B)** of VEGFR2 membrane protein expression in HUVEC and 4T1 cells. **(C)** Confocal microscope images of HUVEC and 4T1 cells incubation with VEGFR2-PEG-HSNs-Fe. **(D)** FCM images of HUVEC and 4T1 cells in different groups (simple cells, targeted competition, non-targeted and targeted groups).

The expression of VEGFR2 protein in HUVEC and 4T1 cells was detected by Western blot. The results showed that the content of VEGFR2 protein in HUVEC cells (63%) was significantly higher than that in 4T1 cells (19%) (**Figures 3A, B** **)**.

After (PE) VEGFR2-PEG-HSNs-Fe NPs were incubated with VEGFR2 overexpressed HUVEC cells, red fluorescence was observed along the cell membrane in the LSCM images (**Figure 3C**), while there was no red fluorescence on the membrane of 4T1 cells. These results demonstrated that VEGFR2-PEG-HSNs-Fe NPs were specifically bind to VEGFR2 protein. It was precisely because VEGFR2 was a tyrosine kinase transmembrane protein distributed in the cell membrane which causes the specific binding of PE fluorescent labeled VEGFR2-PEG-HSNs-Fe NPs to VEGFR2 protein often occurring on the cell surface. Red fluorescence was not observed in non-targeted (**Supplemental 1**), competition (**Supplemental 2**), and pure cells group (**Supplemental 3**).

Quantization results of FCM results were consistent with the qualitative observation of LSCM. FCM images (**Figure 3D**) showed that the most obvious fluorescence intensity shift appeared in the targeted group of HUVEC cells because of their high expression of VEGFR2 protein. HUVEC cells carried a large amount of fluorescein PE through antigen–antibody reaction after incubation with (PE) VEGFR2-PEG-HSNs-Fe NPs. The fluorescence intensity of HUVEC cells was shifted when detected by FCM.

The competition group was designed to verify that the fluorescence on the cells was derived from the combination of VEGFR2 protein and (PE) anti-VEGFR2 antibody. VEGFR2 binding sites on the surface of the cells were occupied by pretreated anti-VEGFR2 antibody. When cultured with (PE)VEGFR2-PEG-HSNs-Fe NPs, it could not bind to cells due to the lack of binding sites, thus inhibiting binding. As a consequence, no fluorescence was observed on LSCM pictures, and no obvious shift was shown on FCM images. Due to the lack of protein that could bind to cells, pure cells and non-targeted groups of HUVEC cells only presented their own fluorescence.

The binding rate of HUVEC cells to (PE) VEGFR2-PEG-HSNs-Fe was the highest (74.80 ± 2.21%), which was significantly higher than that of pure cells, competition, and non-targeted group (4.37 ± 0.63%, 7.11 ± 0.52%, 5.92 ± 0.31%, P < 0.05). Because 4T1 cells did not express the corresponding VEGFR2 protein, the binding rate of this cell group was very low. The binding rate of (PE) VEGFR2-PEG-HSNs-Fe NPs to 4T1 cells was 5.29 ± 0.36%, and there was no difference between the groups (P > 0.05). All these results confirmed that VEGFR2-PEG-HSNs-Fe NPs had a high connection rate with the target cells, which laid a foundation for US imaging *in vivo*.

### Biocompatibility

At present, the biosafety problem of new targeted nano UCA is widespread, which also limits their clinical application. It had been confirmed that SiO_2_ NPs had certain toxicity to cells (28, 29), and the toxicity was affected by various factors, including particle size, surface structure, concentration, contact time, and target cells (30, 31). PEG-HSNs-Fe NPs had been proved less toxic to HUVEC cells and monocyte macrophages in previous studies (32). The structure of VEGFR2-PEG-HSNs-Fe NPs in this study was more complicated because anti-VEGFR2 antibody was used to target re-modification on PEG-HSNs-Fe NPs. Therefore, it was necessary to conduct toxicity tests.

**Figure 4:**
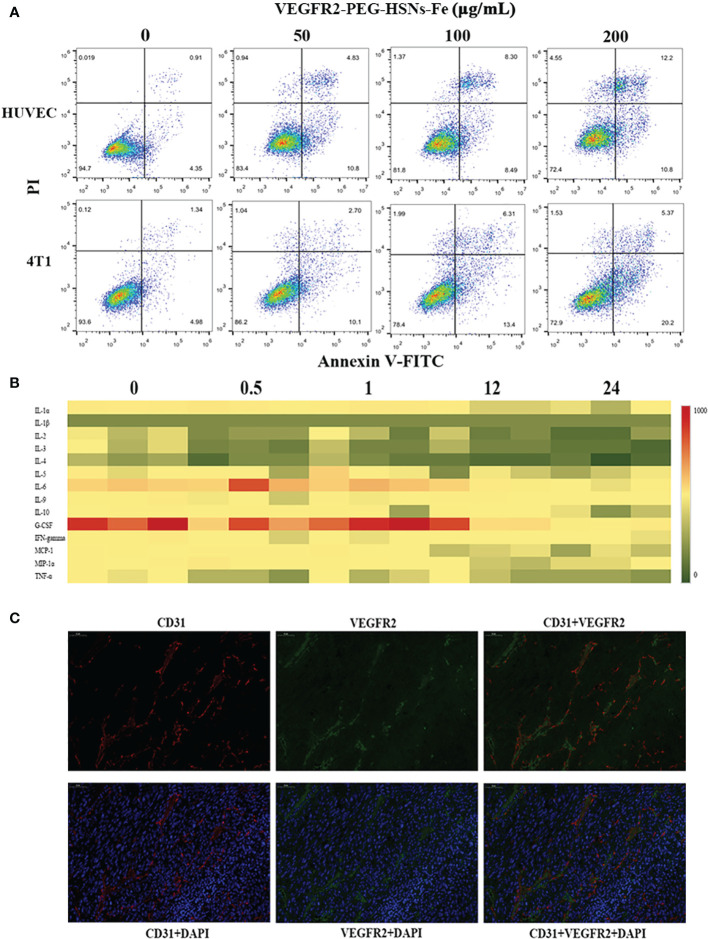
**(A)** FCM images of HUVEC and 4T1 cells at different concentrations of VEGFR2-PEG-HSNs-Fe (50, 100, 200 μg/ml) and PBS for 24 h. **(B)** Heat map of main serum immune factors in nude mice with breast tumor injected with VEGFR2-PEG-HSNs-Fe at different time (0, 0.5, 1, 12, 24). **(C)** Immunofluorescence staining images of 4T1 tumor tissue slices (magnification: ×400). CD31 was labeled by red fluorescence, VEGFR2 by green fluorescence, and tumor nucleus by blue fluorescence.

In *in vitro* toxicity test, cells were detected by annexin-V FITC/PI kit after being incubated with different concentrations of VEGFR2-PEG-HSNs-Fe NPs for 24 h. The results (**Figure 4A**) showed that VEGFR2-PEG-HSNs-Fe NPs mainly led to early apoptosis of 4T1 cells, while it mainly occurred in HUVEC cells in the middle and late stages, but the cell survival rate was still high. With the increased concentration of VEGFR2-PEG-HSNs-Fe NPs, the survival rate of cells decreased gradually; however, it still remained above 80%.

*In vivo* toxicity test included two parts: blood indexes and immune factors. The blood indexes of mice which could reflect the function of main organs were detected after injection of VEGFR2-PEG-HSNs-Fe NPs for 24 h. According to the results shown in **Supplemental 4**, the main blood cells (white blood cells, monocytes, lymphocytes, red blood cells, hemoglobin, mean red blood cell volume, red blood cell volume, mean red blood cell hemoglobin concentration and platelets) and biochemical indexes (AST, ALT, ALP, TBIL, DBIL, CREA, BUN) were all within normal range. To evaluate the effect of VEGFR2-PEG-HSNs-Fe NPs on immune factors, we adopted liquid suspension protein chip to detect the immune factors in mice. The main advantages of this high-throughput biochip included high accuracy and wide range of detection items. It could be used to detect cytokines, antibodies, and microorganisms (33, 34). Heat map showed that there was no obvious immune response in nude mice bearing breast cancer after injection (**Figure 4B**). These data indicated that VEGFR2-PEG-HSNs-Fe NPs had good biocompatibility, and it could be used as a safe nano target UCAs in animal experiments.

### US Imaging *In Vivo*

#### Nude Mice Models

The subcutaneous xenograft tumor was established by injecting 4T1 cell suspension to simulate the microenvironment of mouse breast cancer. It showed a round mass protruding from the skin with an uneven surface. During dissection, it was found that the tumor adhered to the surrounding tissue without obvious boundary, and it was difficult to separate. The gross specimens were dark red, fishy, and tough.

H&E staining sections showed uneven distribution of tumor cells. The size and morphology of cells were different. Moreover, the degree of differentiation was very poor, with large and deep staining nuclei, indicating obvious atypia (**Supplemental 5**). The immunohistochemical staining results showed that cells were dark brown and arranged regularly (**Supplemental 6**), and immunofluorescence staining images (**Figure 4C**) displayed that the blue fluorescence was the tumor nucleus. CD31, as a tumor vascular endothelial marker, showed red fluorescence, and VEGFR2 showed green fluorescence. Red and green fluorescence overlapped on the fusion images, which indicated that CD31 and VEGFR2 were co-localized. This demonstrated that VEGFR2 protein was expressed on endothelial cells of subcutaneous xenograft tumor.

#### US Targeted Imaging *In Vivo*

**Figure 5:**
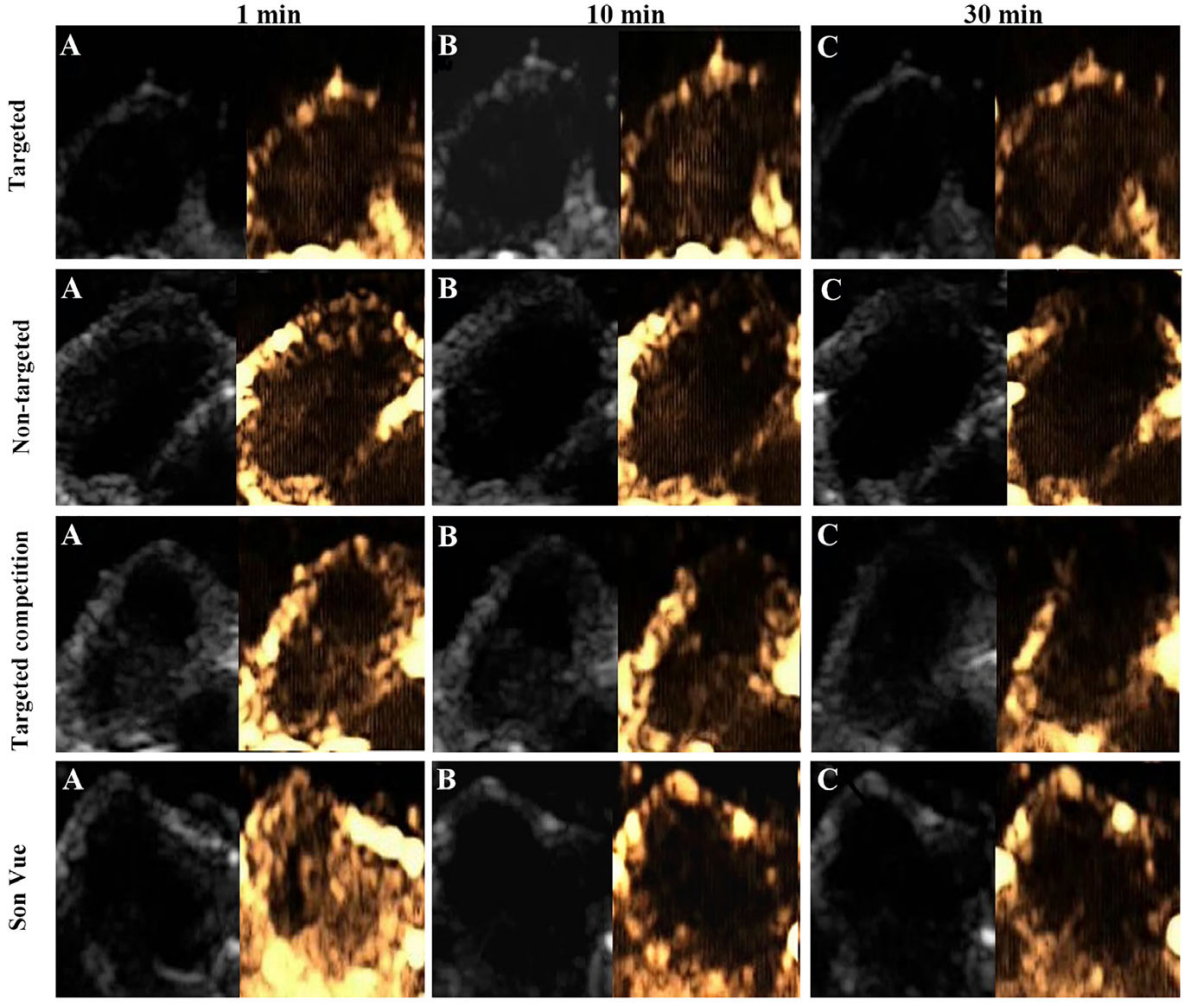
Ultrasound images (B-mode and CEUS) of the targeted group in nude mice with breast cancer (4T1): **(A)** 1 min after injection, **(B)** 10 min after injection, **(C)** 30 min after injection.

Ultrasound targeted imaging of tumor-bearing nude mice was performed by B and CEUS dual mode imaging. **Figure 5** showed that the ultrasonic signal of tumor area was significantly enhanced, and the whole tumor was covered at 1 min after injection of VEGFR2-PEG-HSNs-Fe NPs. It suggested that VEGFR2-PEG-HSNs-Fe NPs had good enhanced US imaging performance. Similarly, ultrasound signal enhancement was also observed in the tumor area in the non-targeted, targeted competition and SonoVue group at 1 min after injection. This indicated that both micron SonoVue and the prepared nano UCAs had ultrasonic imaging ability. However, the enhanced signal intensity of SonoVue group was significantly higher than those of the other three groups. We speculated that this was mainly due to the obvious correlation between the signal intensity and the particle size of ultrasound contrast agent. The particle size of SonoVue was micron, while the other three contrast agents were nano scale.

With the prolongation of imaging time, the signal intensity of tumor in the targeted group was still higher than those of the groups at 1 min, while the signal intensity decreased significantly in the non-targeted, competition, and SonoVue groups. After 30 min, the enhanced ultrasonic signal still could be found in the tumor area in the targeted group, while the other three groups showed obvious hypo-perfusion or no perfusion. Quantitative comparison of the ultrasound signal intensity of different UCAs in tumor area at set time points was shown in the **Supplemental 7**.

The reason for this phenomenon could be explained by the fact that some of VEGFR2-PEG-HSNs-Fe NPs reached the tumor neovascularization through blood circulation and were firmly bonded to the vascular endothelial cells with overexpression of VEGFR2 protein. Specific binding between the antigen and the antibody made the concentrations of VEGFR2-PEG-HSNs-Fe NPs remain relatively stable for a certain period of time. Some NPs could pass through the endothelial cell gap of tumor tissue and accumulated continuously, which caused the concentration of VEGFR2-PEG-HSNs-Fe NPs to increase. Therefore, the targeted group could maintain a good ultrasound enhanced imaging effect or a long period of time. The enhanced signals of the other three groups appeared at the beginning of UCA injection. However, due to lack of specific binding sites, most of UCAs gradually moved out of the tumor through the blood circulation. Even the nano UCAs might stay in the tumor for a short period of time.

The results of these experiments indicated that VEGFR2-PEG-HSNs-Fe NPs prepared in this study had good specific targeting property and imaging ability, which could be used for evaluation of MWA.

#### Evaluation of MWA

The purpose of tumor thermal ablation was to obtain good local therapeutic effect. Imaging evaluation should be performed immediately after operation. If incomplete ablation was detected in time, supplementary ablation could be performed. CEUS was a common method to evaluate the efficacy after ablation. In this study, dual mode (B and CEUS) image was used to evaluate the therapeutic effect of MWA on tumor-bearing nude mice. There was no significant difference in tumor size pre-ablation, including maximum diameter and volume, between the targeted group and SonoVue group (**Table 1**) (P > 0.05).

**Table 1:** Changes of 4T1 breast tumors in nude mice before and after MWA.

Groups	Tumors	Pre MWA	Pro MWA
**Targeted group**	Maximum diameter (mm)	7.73 ± 1.07	8.43 ± 1.01^*^
	Volume (mm^3^)	124 ± 34.05	171.23 ± 33.18^&^
**Sono Vue**	Maximum diameter (mm)	7.53 ± 0.78	7.90 ± 0.82^#^
	Volume (mm^3^)	116.07 ± 22.26	142.99 ± 27.90^^^

The significance of the difference between before and after ablation (^*&#^^P > 0.05).

**Figure 6:**
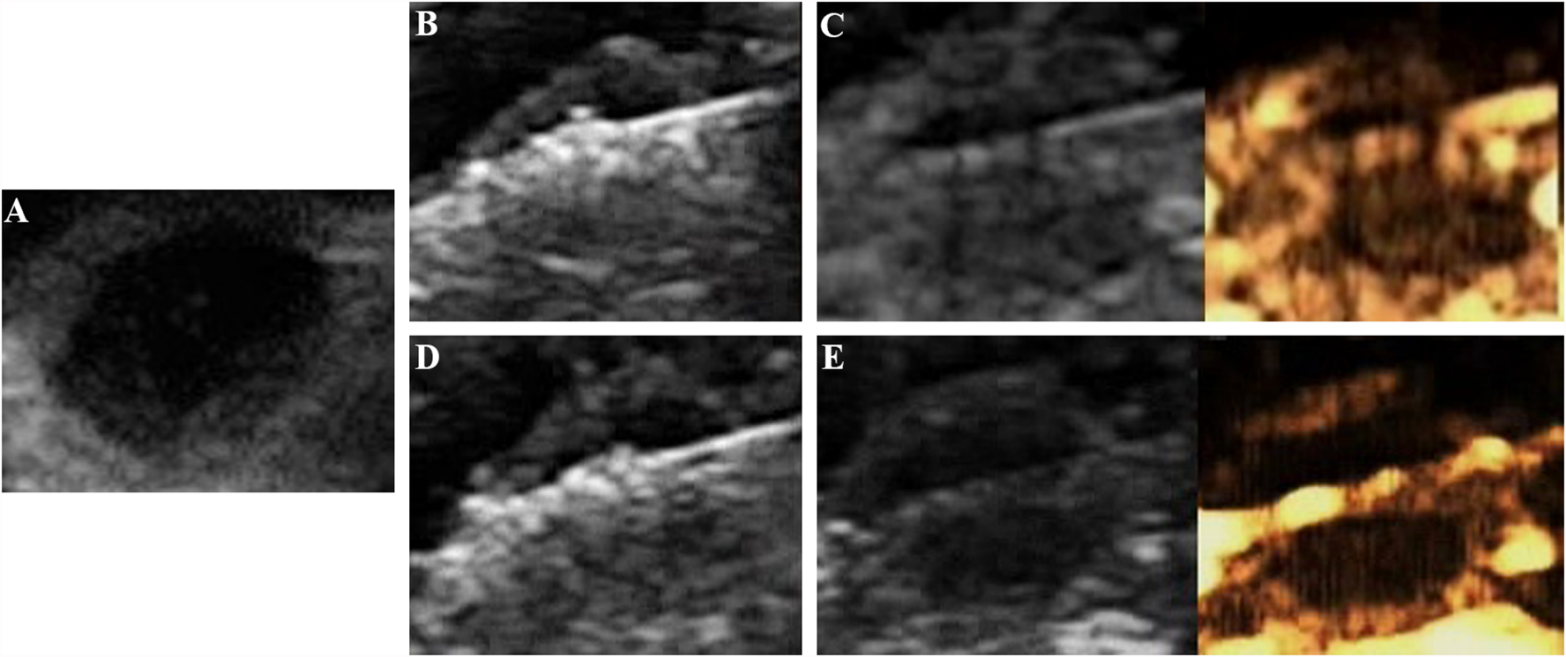
Evaluation of the breast cancer (4T1) in nude mice by the targeted UCA after MWA: **(A)** before MWA, **(B)** hyperechoic region surrounding the antenna caused by vaporization, **(C)** contrast agent perfusion was observed in the periphery of the tumor after ablation. **(A)** complementary ablation: **(D)** a subsequent ablation procedure, **(E)** CEUS confirmed that the second ablation was complete.

**Figure 7:**
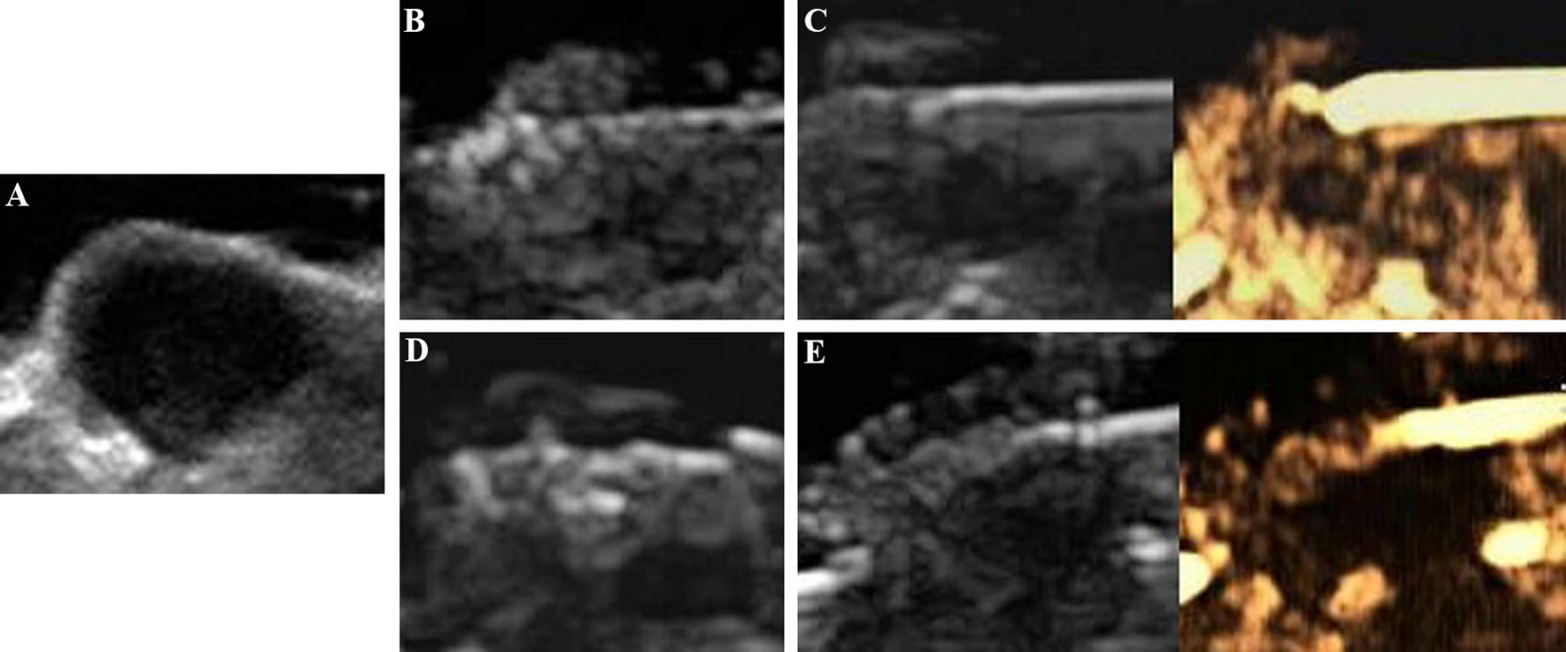
Evaluation of the breast cancer (4T1) in nude mice by SonoVue after MWA: **(A)** before MWA, **(B)** the ablation process, **(C)** contrast agent perfusion was detected in the periphery of the tumor after MWA. A secondary ablation: **(D)** a complementary ablation, **(E)** It was confirmed by CEUS that the complementary ablation was complete.

All tumors were correctly located and successfully ablated according to the preoperative plan. After the gas around the tumor tissue subsided, CEUS was performed by VEGFR2-PEG-HSNs-Fe NPs (**Figure 6**) and SonoVue (**Figure 7**) respectively. Contrast agent perfusion was found on the edge of the lesion. This provided clues for incomplete ablation, suggesting the possibility of residual tumor. After re-ablation and evaluation, no obvious perfusion was found, which proved that the tumors in the two groups were completely ablated. It could be seen from **Table 1** that the maximum diameter and volume of the ablation zone were larger than pre-ablation, but the difference was not statistically significant (P > 0.05). This showed that these two UCAs could meet the basic requirements in the evaluation of curative effect after tumor ablation.

Compared with SonoVue, the ablation time of VEGFR2-PEG-HSNs-Fe NP group (19.00 ± 2.00 min) was slightly shorter than that of SonoVue (25.00 ± 2.00 min), and the difference was statistically significant (P < 0.05). We speculated that this might be due to the relatively rapid regression of SonoVue, although it could also show residual lesions. However, after the ablation of the residual lesions, it was necessary to inject contrast agents for re-evaluation, which would increase the procedure and prolong the ablation time. In contrast, VEGFR2-PEG-HSNs-Fe NPs, a nano-targeted contrast agent, had good targeting stability, which made the enhancement duration longer. The residual lesions could be ablated again and evaluated continuously without repeated injection. In addition, we found that the signal intensity of SonoVue group was significantly higher than that in VEGFR2-PEG-HSNs-Fe group. It was also consistent with the experimental results of targeted imaging of tumor-bearing nude mice *in vivo*.

The above experimental results showed that UCAs could evaluate the ablation effect after MWA and guided the supplementary ablation to achieve complete ablation. Both VEGFR2-PEG-HSNs-Fe NPs and SonoVue had the potential to evaluate the efficacy of tumor ablation. Furthermore, the targeted contrast agents VEGFR2-PEG-HSNs-Fe could reduce the whole ablation time.

Still, this study has some limitations. First of all, the modification of the antibody had only been evaluated by qualitative observation and had not been quantitatively tested. Second, the sample size of animal experiments was small, which made data analysis prone to certain errors. Third, when the UCAs were used for ablation assessment, only the image analysis was performed; the pathological examination of the tumor tissue after ablation to verify the effectiveness of the UCAs for the ablation assessment was not performed. Fourth, although the results showed that the imaging time of VEGFR2-PEG-HSNs-Fe NPs was prolonged, which could detect residual tumor and avoid incomplete ablation, due to the small volume and special subcutaneous position of tumors, the precise measurement of the residual area was limited. The ability of different UCAs to detect residual tumors was not compared in detail. Therefore, the clinical significance of nano targeted UCAs for incomplete ablation should be furtherly studied.

## Conclusion

In summary, VEGFR2-PEG-HSNs-Fe NPs were developed as a new nano targeted UCAs for specifically targeting tumor tissue. It could be used as a means to evaluate the curative effect after ablation and shorten the operation time. This provided a new method for post-ablation evaluation and might open up a new field for targeted nano UCA applications.

## Data Availability Statement

The original contributions presented in the study are included in the article/**Supplementary Material**. Further inquiries can be directed to the corresponding authors.

## Ethics Statement

The animal study was reviewed and approved by Ethics Committee and Animal Care Committee of Ruijin Hospital, Shanghai Jiao Tong University School of Medicine.

## Author Contributions

XL and WZ carried out the preparation of materials and imaging experiments *in vivo*. XL and SX wrote the main manuscript. SX studied the cell experiment *in vitro*. RJ completed the preparation and characterization of materials. WWZ and WZ were involved to experiments design, literature study and data analysis. All authors contributed to the article and approved the submitted version.

## Funding

This work was supported by grants from Foundation of Ruijin Hospital/Lu Wan Branch, Shanghai Jiao Tong University School of Medicine (YQA202001) and National Natural Science Foundation of China (No. 81701710).

## Conflict of Interest

The authors declare that the research was conducted in the absence of any commercial or financial relationships that could be construed as a potential conflict of interest.

## References

[1] Zhou W Ni X Xu S Zhang L Chen Y Zhan W. Ultrasound-Guided Laser Ablation Versus Surgery for Solitary Papillary Thyroid Microcarcinoma: A Retrospective Study. Int J Hyperthermia (2019) 36(1):897–904. 10.1080/02656736.2019.1649475 31464140

[2] Mauri G Cova L Tondolo T Ierace T Baroli A Di Mauro E. Percutaneous Laser Ablation of Metastatic Lymph Nodes in the Neck From Papillary Thyroid Carcinoma: Preliminary Results. J Clin Endocrinol Metab (2013) 98(7):E1203–7. 10.1210/jc.2013-1140 23666969

[3] Kim JH Yoo WS Park YJ Park DJ Yun TJ Choi SH. Efficacy and Safety of Radiofrequency Ablation for Treatment of Locally Recurrent Thyroid Cancers Smaller Than 2 Cm. Radiology (2015) 276(3):909–18. 10.1148/radiol.15140079 25848897

[4] Yue W Wang S Yu S Wang B. Ultrasound-Guided Percutaneous Microwave Ablation of Solitary T1N0M0 Papillary Thyroid Microcarcinoma: Initial Experience. Int J Hyperthermia (2014) 30(2):150–7. 10.3109/02656736.2014.885590 24571178

[5] Wang J Liang P Yu J Yu MA Liu F Cheng Z. Clinical Outcome of Ultrasound-Guided Percutaneous Microwave Ablation on Colorectal Liver Metastases. Oncol Lett (2014) 8(1):323–6. 10.3892/ol.2014.2106 PMC4063642 24959270

[6] Cheng Z Yu X Han Z Liu F Yu J Liang P. Ultrasound-Guided Hydrodissection for Assisting Percutaneous Microwave Ablation of Renal Cell Carcinomas Adjacent to Intestinal Tracts: A Preliminary Clinical Study. Int J Hyperthermia (2018) 34(3):315–20. 10.1080/02656736.2017.1338362 28641464

[7] Moussa AM Ziv E Solomon SB Camacho JC. Microwave Ablation in Primary Lung Malignancies. Semin Intervent Radiol (2019) 36(4):326–33. 10.1055/s-0039-1700567 PMC6823043 31680724

[8] Kong P Pan H Yu M Chen L Ge H Zhu J. Insufficient Microwave Ablation-Induced Promotion of Distant Metastasis is Suppressed by β-Catenin Pathway Inhibition in Breast Cancer. Oncotarget (2017) 8(70):115089–101. 10.18632/oncotarget.22859 PMC5777756 29383144

[9] Zhang N Li H Qin C Ma D Zhao Y Zhu W. Insufficient Radiofrequency Ablation Promotes the Metastasis of Residual Hepatocellular Carcinoma Cells Via Upregulating Flotillin Proteins. J Cancer Res Clin Oncol (2019) 145(4):895–907. 10.1007/s00432-019-02852-z 30820716 PMC6435628

[10] Ricci P Cantisani V Drudi F Pagliara E Bezzi M Meloni F. Is Contrast-Enhanced US Alternative to Spiral CT in the Assessment of Treatment Outcome of Radiofrequency Ablation in Hepatocellular Carcinoma? Ultraschall Med (2009) 30(3):252–8. 10.1055/s-2008-1027727 19280552

[11] Nishigaki Y Hayashi H Tomita E Suzuki Y Watanabe N Watanabe S. Usefulness of Contrast-Enhanced Ultrasonography Using Sonazoid for the Assessment of Therapeutic Response to Percutaneous Radiofrequency Ablation for Hepatocellular Carcinoma. Hepatol Res (2015) 45(4):432–40. 10.1111/hepr.12370 24917381

[12] Qian GJ Wang N Shen Q Sheng YH Zhao JQ Kuang M. Efficacy of Microwave Versus Radiofrequency Ablation for Treatment of Small Hepatocellular Carcinoma: Experimental and Clinical Studies. Eur Radiol (2012) 22(9):1983–90. 10.1007/s00330-012-2442-1 22544225

[13] Wu JY Chen MH Yang W Lin SZ Wu W Yin SS. Role of Contrast Enhanced Ultrasound in Radiofrequency Ablation of Metastatic Liver Carcinoma. Chin J Cancer Res (2012) 24(1):44–51. 10.1007/s11670-012-0044-8 23359761 PMC3555259

[14] Knowles JA Heath CH Saini R Umphrey H Warram J Hoyt K. Molecular Targeting of Ultrasonographic Contrast Agent for Detection of Head and Neck Squamous Cell Carcinoma. Arch Otolaryngology-Head Neck Surg (2012) 138(7):662–8. 10.1001/archoto.2012.1081 PMC3417358 22801891

[15] Baron Toaldo M Salvatore V Marinelli S Palamà C Milazzo M Croci L. Use of VEGFR-2 Targeted Ultrasound Contrast Agent for the Early Evaluation of Response to Sorafenib in a Mouse Model of Hepatocellular Carcinoma. Mol Imaging Biol (2015) 17(1):29–37. 10.1007/s11307-014-0764-x 25082536

[16] Wischhusen J Wilson KE Delcros JG Molina-Peña R Gibert B Jiang S. Ultrasound Molecular Imaging as a non-Invasive Companion Diagnostic for Netrin-1 Interference Therapy in Breast Cancer. Theranostics (2018) 8(18):5126–42. 10.7150/thno.27221 PMC6217066 30429890

[17] Klibanov AL. Molecular Imaging With Targeted Ultrasound Contrast Microbubbles. Ernst Schering Res Found Workshop (2005) 49:171–91. 10.1007/3-540-26809-X_10 15524217

[18] Wang S Hossack JA Klibanov AL. Targeting of Microbubbles: Contrast Agents for Ultrasound Molecular Imaging. J Drug Target (2018) 26(5–6):420–34. 10.1080/1061186X.2017.1419362 PMC6319889 29258335

[19] Lanza GM Abendschein DR Hall CS Scott MJ Scherrer DE Houseman A. In Vivo Molecular Imaging of Stretch Induced Tissue Factor in Carotid Arteries With Ligand-Targeted Nanoparticles. J Am Soc Echocardiogr (2000) 13(6):608–14. 10.1067/mje.2000.105840 10849515

[20] Maeda H Bharate GY Daruwalla J. Polymeric Drugs for Efficient Tumor-Targeted Drug Delivery Based on EPR-Effect. Eur J Pharm Biopharm (2009) 71(3):409–19. 10.1016/j.ejpb.2008.11.010 19070661

[21] Yao KX Zeng HC. Simultaneous Chemical Modification and Structural Transformation of Stöber Silica Spheres for Integration of Nanocatalysts. Chem Materials (2011) 24(1):140–8. 10.1021/cm2030119

[22] Yu L Chen Y Wu M Cai X Yao H Zhang L. “Manganese Extraction” Strategy Enables Tumor-Sensitive Biodegradability and Theranostics of Nanoparticles. J Am Chem Soc (2016) 138(31):9881–94. 10.1021/jacs.6b04299 27441571

[23] Yec CC Zeng HC. Nanobubbles Within a Microbubble: Synthesis and Self-Assembly of Hollow Manganese Silicate and its Metal-Doped Derivatives. ACS nano (2014) 8(6):6407–16. 10.1021/nn501948h 24878224

[24] Deshpande N Needles A Willmann JK. Molecular Ultrasound Imaging: Current Status and Future Directions. Clin Radiol (2010) 65(7):567–81. 10.1016/j.crad.2010.02.013 PMC3144865 20541656

[25] Maeda H Fang J Inutsuka T Kitamoto Y. Vascular Permeability Enhancement in Solid Tumor: Various Factors, Mechanisms Involved and Its Implications. Int Immunopharmacol (2003) 3(3):319–28. 10.1016/S1567-5769(02)00271-0 12639809

[26] Maeda H Sawa T Konno T. Mechanism of Tumor-Targeted Delivery of Macromolecular Drugs, Including the EPR Effect in Solid Tumor and Clinical Overview of the Prototype Polymeric Drug SMANCS. J Control Release (2001) 74(1-3):47–61. 10.1016/S0168-3659(01)00309-1 11489482

[27] Oeffinger BE Wheatley MA. Development and Characterization of a Nano-Scale Contrast Agent. Ultrasonics (2004) 42(1-9):343–7. 10.1016/j.ultras.2003.11.011 15047309

[28] De Marzi MC Saraceno M Mitarotonda R Todone M Fernandez M Malchiodi EL. Evidence of Size-Dependent Effect of Silica Micro- and Nano-Particles on Basal and Specialized Monocyte Functions. Ther Deliv (2017) 8(12):1035–49. 10.4155/tde-2017-0053 29125067

[29] Napierska D Quarck R Thomassen LC Lison D Martens JA Delcroix M. Amorphous Silica Nanoparticles Promote Monocyte Adhesion to Human Endothelial Cells: Size-Dependent Effect. Small (2013) 9(3):430–8. 10.1002/smll.201201033 23042701

[30] Li YB Zhou W Yu YB Duan JC Sun ZW. Cytotoxicity and Oxidative Damage Effect of Silica Nanoparticles on Vascular Endothelial Cells. J Jilin Univ (2014) 40(3):476–81. 10.13481/j.1671-587x.20140303

[31] Berg JM Romoser AA Figueroa DE Spencer West C Sayes CM. Comparative Cytological Responses of Lung Epithelial and Pleural Mesothelial Cells Following In Vitro Exposure to Nanoscale Sio2. Toxicol Vitro (2013) 27(1):24–33. 10.1016/j.tiv.2012.09.002 22985735

[32] Laranjeira M Shirosaki Y Yoshimatsu YS Miyazaki T Monteiro FJ. Enhanced Biosafety of Silica Coated Gadolinium Based Nanoparticles. J Mater Sci Mater Med (2017) 28(3):46. 10.1007/s10856-017-5855-1 28161832

[33] Koczera P Appold L Shi Y Liu M Dasgupta A Pathak V. PBCA-Based Polymeric Microbubbles for Molecular Imaging and Drug Delivery. J Control Release (2017) 259:128–35. 10.1016/j.jconrel.2017.03.006 PMC5528138 28279799

[34] Huang P Rong P Jin A Yan X Zhang MG Lin J. Dye-Loaded Ferritin Nanocages for Multimodal Imaging and Photothermal Therapy. Adv Mater (2014) 26(37):6401–8. 10.1002/adma.201400914 PMC4215197 25123089

