# Supplementary material for: Evaluation of Microwave Ablation in 4T1 Breast Tumor by a Novel VEFGR2 Targeted Ultrasound Contrast Agents
Source: Front Oncol. 2021 Jul 20;11:690152. doi: 10.3389/fonc.2021.690152 (PMC8329532; doi:10.3389/fonc.2021.690152)
Supplement: Supplementary file 1 [file DataSheet_1.docx]

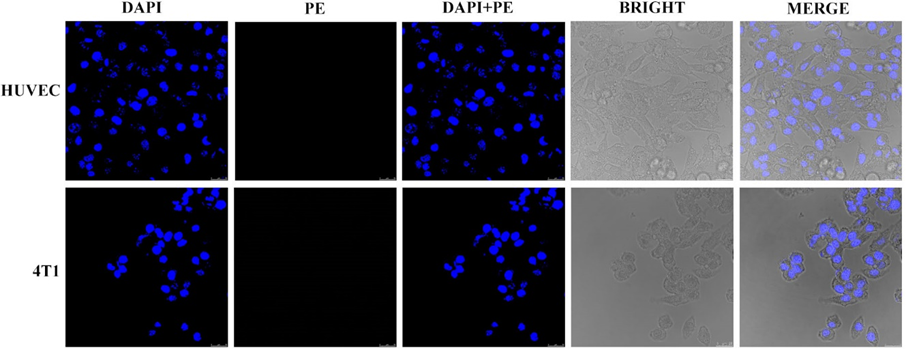


Supplement 1. LSCM images of HUVEC and 4T1 cells incubated with PEG-HSNs-Fe NPs in non-targeted group.


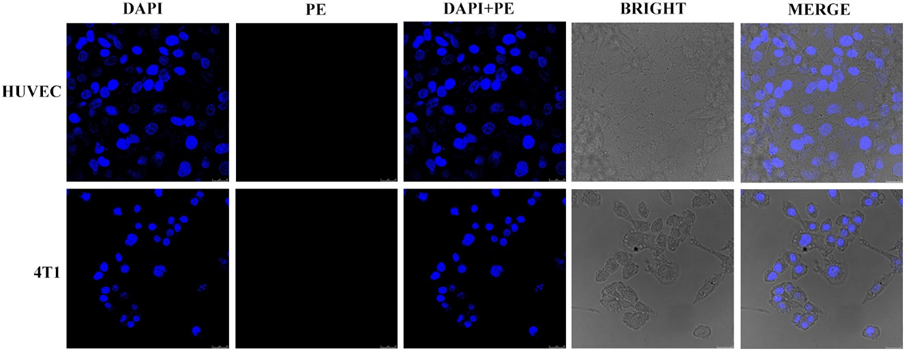


Supplement 2. LSCM images of HUVEC and 4T1 cells in targeted competition group.


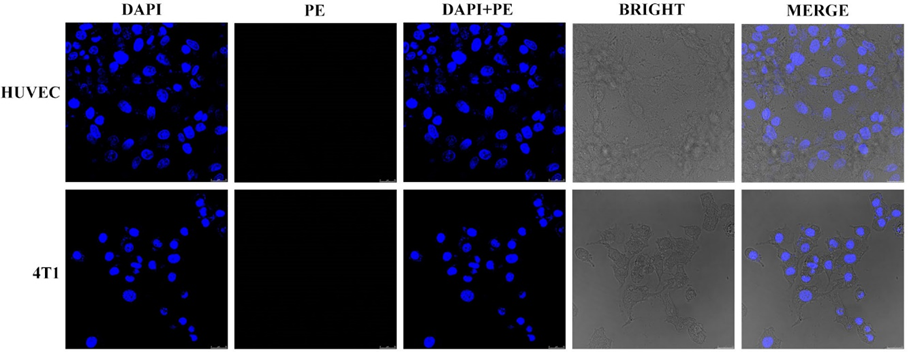


Supplement 3. LSCM images of HUVEC and 4T1 cells in pure cells group.


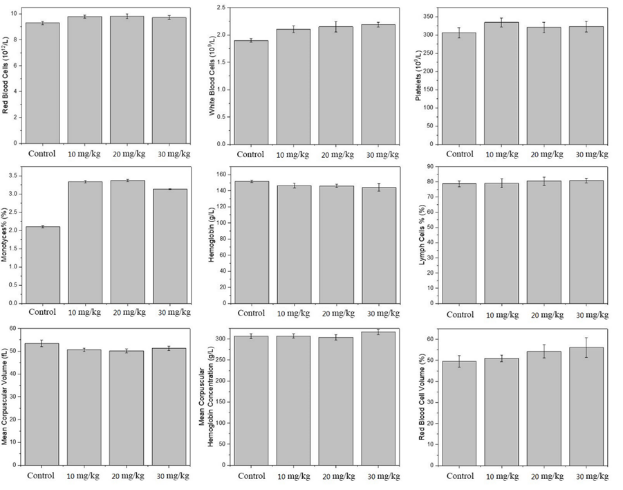

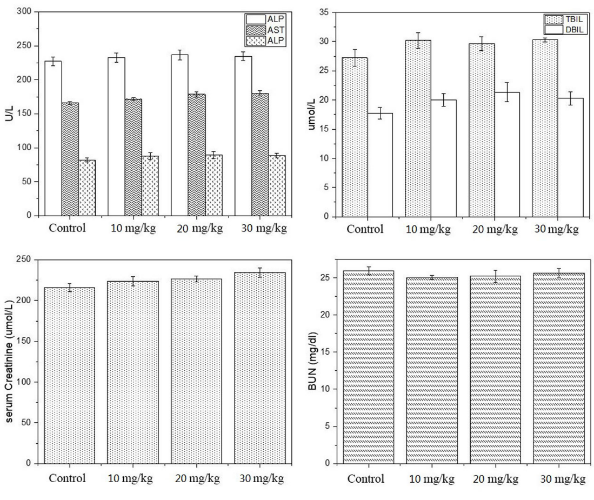


Supplement 4. The blood indexes after injection of VEGFR2-PEG-HSNs-Fe NPs.


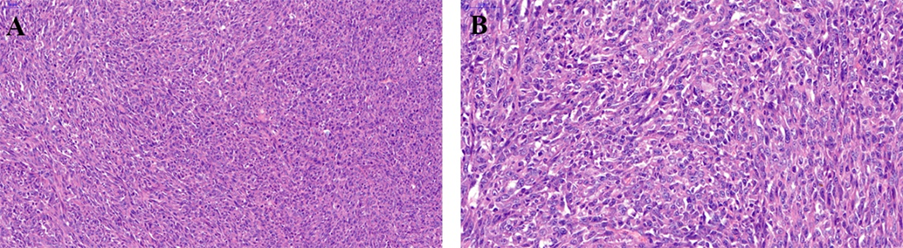
Supplement 5. Hematoxylin and eosin-stained images of tumor tissue slices.

(A) magnification: ×200; (B) magnification: ×400.


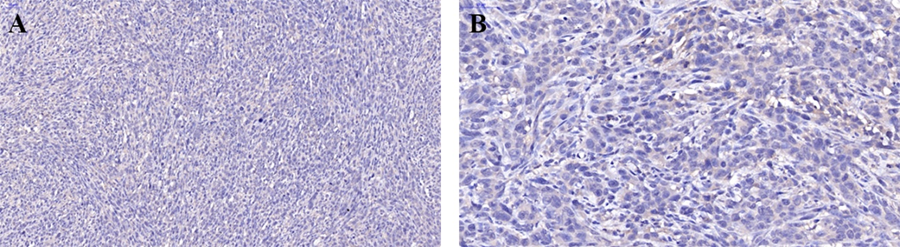


Supplement 6. Immunohistochemical images of tumor tissue slices. (A) magnification: ×200; (B) magnification: ×400.


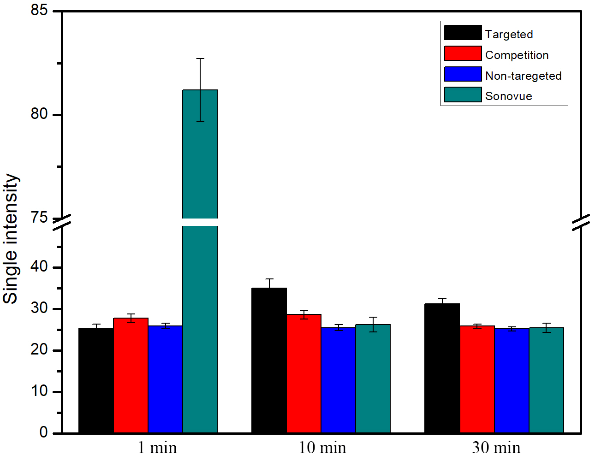


Supplement 7. Comparison of signal intensity of different UCAs in breast tumor-bearing nude mice at different time points (1, 10, 30 minutes).
